# Long-Term Dentin Bonding Performance of Universal Adhesives: The Effect of HEMA Content and Bioactive Resin Composite

**DOI:** 10.3390/jfb15120379

**Published:** 2024-12-16

**Authors:** Di Wu, Ye Yao, Carolina Cecilia Cifuentes-Jimenez, Hidehiko Sano, Pedro Álvarez-Lloret, Monica Yamauti, Atsushi Tomokiyo

**Affiliations:** 1 Department of Restorative Dentistry, Graduate School of Dental Medicine, Hokkaido University, Sapporo 060-8586, Japan; wudi0526@den.hokudai.ac.jp (D.W.); yaoye@den.hokudai.ac.jp (Y.Y.); sano@den.hokudai.ac.jp (H.S.); tomokiyo@den.hokudai.ac.jp (A.T.); 2 Department of Stomatology, Faculty of Dentistry, University of Granada, Campus de Cartuja, s/n, 18011 Granada, Spain; carolinaccj@correo.ugr.es; 3 Department of Geology, Faculty of Geology, University of Oviedo, Campus de Llamaquique, s/n, 33005 Oviedo, Spain; pedroalvarez@uniovi.es; 4 Department of Biomedical and Applied Sciences, School of Dentistry, Indiana University Indianapolis, Indianapolis, IN 46202, USA

**Keywords:** universal adhesive, S-PRG fillers, HEMA, degree of conversion, water sorption and solubility, ions release, bond strength

## Abstract

This study investigated the effects of resin composites (RCs) containing surface pre-reacted glass ionomer (S-PRG) filler on the dentin microtensile bond strength (μTBS) of HEMA-free and HEMA-containing universal adhesives (UAs). Water sorption (WS) and solubility (SL), degree of conversion (DC), and ion release were measured. The UAs BeautiBond Xtreme (BBX; 0% HEMA), Modified Adhesive-1 (E-BBX1; 5% HEMA), Modified Adhesive-2 (E-BBX2; 10% HEMA), and two 2-step self-etch adhesives (2-SEAs): FL-BOND II (FBII; with S-PRG filler) and silica-containing adhesive (E-FBII) were used. Teeth were restored with Beautifil Flow Plus F00 with S-PRG filler (BFP) and flowable resin composite with silica filler (E-BFP). μTBS was evaluated after 24 h and 6 months of water storage. WS and SL measurement followed ISO 4049:2019; spectroscopy measured DC; ICP-MS evaluated ion release. BBX and FBII presented the highest DC. The adhesives did not comply with the WS ISO requirements, but the bonding resin of 2-SEAs complied with the SL threshold. BFP released more ions than E-BFP. BFP positively affected the μTBS of UAs, regardless of HEMA concentration after 24 h, comparable to the 2-SEAs. The 6 months μTBS decrease depended on the adhesive and RC combination. HEMA did not affect the μTBS of UAs, while bioactive resins had a positive impact.

## 1. Introduction

Universal adhesives (UAs) are widely recognized as adhesive systems that can be applied in etch-rinse and self-etch modes to various dental tissues and materials utilized in direct or indirect restorative techniques [1]. Due to their multifunctional features, their composition is more complex than the preceding all-in-one self-etch adhesive systems. When developing single-bottle UAs, an ideal mixture of acrylic resin monomers with differing hydrophilicity and hydrophobicity, solvent water, silane, and functional monomers needed to be blended and function well in combination, ideally polymerized to produce a durable bonded interface [2]. However, achieving an ideal composition is highly technique-sensitive since all adhesive compounds blend, even though they are not always miscible [3].

A functional monomer, 2-hydroxyethyl methacrylate (HEMA), is frequently added to UAs. HEMA is highly soluble in other solvents and water; its hydrophilic and co-solvent nature improves stability and helps to keep hydrophobic and hydrophilic monomers in a homogenous solution by reducing phase separation [4]. Phase separation occurs when the solvent evaporates at moist bonding interfaces, especially when using adhesives like UAs that contain high water concentrations [1]. Moreover, HEMA’s polar characteristics and water-solubility properties increase wettability and promote resin monomer diffusion into the dentin collagen fibril network [5]. However, uncured HEMA also lowers water vapor pressure in the adhesive and may make it more challenging to evaporate during the air-drying step, impairing polymerization [6]. Due to its high hydrophilicity, HEMA readily absorbs water in its uncured and polymerized state [7]. High water sorption can cause a decrease in mechanical properties and contribute to the degradation of dental polymer matrices [5]. A previous investigation has also suggested that HEMA hampers the interactions between the phosphate groups of 10-MDP and hydroxyapatite (HAp), which could undermine the bond strength of UAs containing substantial amounts of HEMA [8]. To increase the durability of the composite restoration, manufacturers and researchers have attempted to reduce the hydrophilicity of UAs by adjusting the concentrations of HEMA to increase the longevity of the composite restoration [9]. UAs could still be considered a one-step adhesive [2].

The so-called controversial bioactivity has been another property addressed in dental restorative materials for a stable dentin-resin bonding [1]. The latest definition of bioactivity from FDI divides the mechanisms into three levels: purely biological, mixed biological/chemical, or strictly chemical (e.g., through ion release from bioactive glass fillers [10]). Recently, significant interest has been drawn to using glass particles that demonstrate various effects by releasing multiple ions [11]; among them, the silanated pre-reacted glass ionomer (S-PRG) filler has been widely used in commercial products. According to the manufacturer, the S-PRG filler is a multilayered, ultrafine glass particle with a SiO_2_ coating on the outer layer, a pre-reacted glass-ionomer phase in the middle, and a glass core that could be released into dental hard tissues and enhance their mineralization [12].

The cured adhesive layers in single-step adhesives may act as semi-permeable membranes that allow water diffusion from the bonded hydrated dentin to the intermixed zone between the adhesive and the composite [13]. A recent study reported that multi-ions released by S-PRG-filled resin composite might permeate tooth substrates through adhesives, hindering tooth demineralization around composite restorations [14]. To combine the advantages of UAs and the properties of S-PRG fillers, new resin-based products have been developed and require investigation. Therefore, this study aimed to investigate the effects of a resin composite (RC) containing S-PRG filler on the dentin bond strength of HEMA-free and HEMA-containing UAs over a period of 6 months. Two different two-step self-etch adhesives (2-SEAs) were used as reference groups. Additionally, the water sorption and solubility of the materials, as well as their degree of conversion, were investigated. The hypotheses were that (1) there would be no significant differences in the degree of conversion of materials; (2) there would be no difference in the water sorption and solubility of adhesive systems and RCs; (3) there would be no difference in the ion release of adhesives and RCs; (4) the storage period, adhesive types, RCs, and their combinations would not affect the dentin bond strength to dentin.

## 2. Materials and Methods

### 2.1. Study Design and Ethical Considerations

This study represents quantitative, qualitative, and prospective in vitro research. The experimental factors were adhesive type (5 levels), restorative resin (2 levels), and storage period (2 levels—only for bond strength). The response variables were degree of conversion, water sorption and solubility, and bond strength. The research protocol and the use of human extracted teeth was approved by the Institution’s Human Research Ethics Committee in the Graduate School of Dental Medicine, Hokkaido University (Protocol #2018/9). The teeth were preserved in an aqueous solution of 0.5% chloramine-T at 4 °C and used within 6 months of extraction.

### 2.2. Degree of Conversion of Adhesives and Resin Composites

The materials used in the study are listed in Table 1, along with their manufacturers, abbreviations, and instructions. A modular confocal Raman spectrograph was used to investigate the degree of conversion (DC) of the adhesives under non-polymerized and polymerized conditions. Raman spectra were obtained using a JASCO NRS-5100 (Jasco Inc., Easton, MD, USA) spectrometer with a charge-coupled detector (1024 × 256 pixels) cooled by a Peltier-effect module. Two drops of each adhesive were poured into a circular Teflon mold (10.0 mm diameter × 4.0 mm depth) and placed under magnification on an XYZ stage. A near-infrared diode laser (785 nm) kept at 500 mW was employed to induce the Raman scattering, focusing the laser beam with a 20× lens (optical microscope). Spectra were acquired in a range between 1000 and 1800 cm^−1^ using an exposure time of 5 s and 10 accumulations with an average spectral resolution of 1.6 cm^−1^. The adhesive samples were analyzed while non-polymerized to obtain the uncured monomer spectra. Subsequently, each adhesive sample was light-activated using an LED curing unit with light irradiance of 1200 mW/cm^2^ (G-Light Prima-II Plus; GC Corp., Tokyo, Japan) following the manufacturer’s instructions, and the polymerized measurements were taken. Instrument calibration was determined before data acquisition by comparison with the silicon standard spectrum to set the reference position at 520 cm^−1^.

Resin composite samples were prepared using a circular bipartite Teflon mold (10.0 mm diameter × 1.0 mm thick) placed on top of a glass slide and analyzed using a Fourier transform infrared (FTIR) JASCO 6200 (Jasco Inc., Easton, MD, USA) spectrometer equipped with a diamond-tip attenuated total reflection (ATR) accessory (ATR Pro ONE; Jasco Inc.). The samples were placed on the ATR glass holder, covering the surface before and after polymerization. All spectra were acquired in absorbance mode between 600 and 4000 cm^−1^ spectral range, with a resolution of 2 cm^−1^ and 124 scan accumulations. Three samples of each uncured adhesive/composite material were analyzed.

The DC values were calculated by determining the area ratio of the absorbance aliphatic C=C at 1638 cm^−1^ and the internal reference peak of aromatic C=C at 1608 cm^−1^ [15]. A region of the spectra between 1590 and 1660 cm^−1^ was selected and baseline corrected; after spectrometric analyses, the DC was calculated as follows:

$$\mathrm{DC}\;(\%{)}_{\mathrm{area}}=[1-\frac{{Cured\;C=C\;1638\;cm}^{-1}/{Cured\;C=C\;1608\;cm}^{-1}}{{Uncured\;C=C\;1638\;cm}^{-1}/{Uncured\;C=C\;1608\;cm}^{-1}}]\times 100$$

The area peak values were resolved using curve-fitting software Peakfit v4.12 (Systat Software, Chicago, IL, USA). The second derivative method was utilized for the measurements of each peak within the spectral region. The degree of smoothing was set at 20% (Savitzky–Golay algorithm), and a mixed Gaussian-Lorentzian function was used to adjust the peak profiles (i.e., curve shape and width). Curve fitting was accepted when r^2^ reached values up to 0.995.

### 2.3. Water Sorption and Solubility

A metal mold (15.0 ± 0.1 mm diameter, 1.0 ± 0.1 mm thick) was used to prepare resin disks (*n* = 6), and a silicone rubber mold (6.0 ± 0.1 mm diameter, 1.0 ± 0.1 mm thick) was used to produce adhesive disks (*n* = 6). The measurement and calculation of water sorption (WS) and solubility (SL) followed the guidelines specified in ISO 4049:2019 [16]. After polymerization, the samples were promptly transferred to a desiccator and moved to a pre-conditioning oven set at 37 °C. All samples were dried in a silica gel desiccator for 22 h at 37 ± 2 °C and 2 h at 23 ± 2 °C. The samples underwent weightings at 24 h intervals until a stable mass (*m*1) was achieved, indicated by a fluctuation of less than 100 μg over 24 h for 3 days. The dimensions of the samples were assessed utilizing a digital caliper, with values rounded to the nearest 0.01 mm, and these measurements were used to calculate the volume (*V*: mm^3^) of each sample. Subsequently, the samples were individually introduced into hermetically sealed glass vials, each containing 10 mL (for adhesive disk) and 20 mL (for resin disk) of deionized water with a pH of 7.2, and maintained at a temperature of 37 °C. Following a storage period of 7 days, the vials were removed from the oven and allowed to equilibrate at ambient temperature for 20 min. The samples were washed using running water and gently wiped using soft absorbent paper. Subsequently, the samples were weighed using an analytical balance, identified as *m*2. After a storage period of 7 days at ambient conditions, the samples were subjected to a drying process within a desiccator that contained newly replenished silica gel. As previously explained, the samples were weighed daily until they attained a stable mass (*m*3). The mass measured after the initial desiccation process (*m*1) was used to compute the mass variation at regular intervals during 7 days of water storage. The kinetics of water absorption during the entire duration of water storage were determined by plotting changes in mass against the storage period. WS and solubility SL values were determined during 7 days of water storage. The calculations were performed using the formulas *WS* = (*m*2 − *m*3)/*V* and *SL* = (*m*1 − *m*3)/*V* [16].

### 2.4. Measurement of Ion Release

The 7-day immersing solutions of adhesives and resin disks from the SL test were used to measure ion release (*n* = 6). The immersion solutions (0.5 mL) were examined using an inductively coupled plasma-mass spectrometer (ICP-MS; 8900 Triple Quadrupole, Agilent Corp, Santa Clara, CA, USA) to quantify the levels of B, Na, Al, Si, and Sr ions. This analysis utilized a pre-established calibration curve that relates the spectrometry values to the standard concentration of multiple elements (XSTC-622; Seishin Trading, Kobe, Japan). The release of F ions was examined using an ion chromatography device (Dionex™ ICS 1600; Thermo Fisher Scientific Inc., Waltham, MA, USA) attached to a high-speed anion chromatography column (TSKgel guard column SuperIC-AHS; Tosoh Corp., Tokyo, Japan). To achieve pH stabilization, 0.7 mM Na_2_CO_3_ buffer (37991-13; Kanto Chemical, Tokyo, Japan) was introduced into the solution. The calibration curve was constructed by diluting the anion mixed standard solution (01849-96, Kanto Chemical, Tokyo, Japan) with ultrapure water [17].

### 2.5. Microtensile Bond Strength (µTBS) and Failure Mode

Eighty sound human molars were randomly selected for the µTBS test, checking for the absence of cavities, fissures, or fractures using a stereoscope (Moticam 1080; Shimadzu Corp., Kyoto, Japan). Flat, occlusal dentin surfaces were exposed using a gypsum model trimmer (Model Trimmer MT 10; J. Morita MFG. Corp., Tokyo, Japan) under water coolant. The surfaces were then checked with a stereoscope to ensure no enamel was remaining on the surfaces. The smear layer was standardized for 60 s using 600-grit silicon carbide paper (Fuji Star Type DDC, Sankyo Rikagaku Co., Saitama, Japan) under high-flowing water polishing. Then, they were randomly divided into five groups according to the dental adhesives: Beautibond Xtreme (BBX), Modified Adhesive 1 (E-BBX1), Modified Adhesive 2 (E-BBX2), FL-Bond II (FBII), silica-containing adhesive (E-FBII), which were applied following the manufacturer’s instructions (Table 1). Each bonded group (*n* = 16) was further divided into two subgroups (*n* = 8) according to the flowable resin composite used (Beautifil Flow Plus F00 [BFP] and silica-containing flowable resin composite [E-BFP]). Each flowable resin composite was applied incrementally (1 mm increment) on dentin surfaces to build up the resin blocks (4 mm thick) and light-cured with a blue LED light-curing unit (G-Light Prima-II Plus; GC Corporation, Tokyo, Japan) with light irradiance of 1200 mW/cm^2^. According to the manufacturer’s instructions, when UAs were utilized, the curing time was 5 s, and the curing time of the bonding resin of the two-step self-etch adhesives was 10 s. The flowable resin composite was light-cured for 10 s per layer. The µTBS test followed the Academy of Dental Materials’ recommendations for the non-trimmed µTBS testing [18]. After 24 h (24 h) of storage in distilled water at 37 °C, all teeth underwent longitudinal sectioning in two perpendicular directions across the bonded interface. A low-speed diamond saw (Isomet^®^ 1000; Buehler Ltd., Lake Bluff, IL, USA) with water cooling was used for this procedure, resulting in beam sticks with a cross-sectional area of about 1 mm^2^. Following the sectioning procedure, half of the beams were immediately subjected to testing, while the other half were stored for 6 months (6 m) in distilled water at 37 °C. According to the specified storage period (24 h or 6 m), each beam was fixed to a jig utilizing a cyanoacrylate glue (Model Repair II Blue; Dentsply-Sankin, Tokyo, Japan) installed in a tabletop testing machine (EZ-S test; Shimadzu Corp., Tokyo, Japan) to be tested under tension at a 1.0 mm/min crosshead speed. The bond strength value of each beam was calculated and expressed in megapascals (MPa). Then, the mean of the bond strength obtained from all beams in each tooth was calculated, and “tooth” was used as the statistical measure. The fractured beams were observed with a stereoscope equipped with a digital camera system (Moticam 1080; Shimadzu Corp., Tokyo, Japan) at a magnification of 100×. Failure modes were categorized as follows: Adhesive failure (A): failure that took place in the adhesive area; cohesive failure in resin composite (CC): failure that occurred within the resin composite; cohesive failure in dentin (CD): failure that occurred within dentin; and mixed failure (M): failure from the adhesive into resin composite and/or dentin area [9]. Additionally, representative beams, after fracture, from each group (*n* = 24) were placed on aluminum stubs, and sputter-coated for 120 s with Pt-Pd using an ion sputtering machine (E-1030; Hitachi Ltd., Tokyo, Japan). The fractured surfaces were observed using a field emission scanning electron microscope (SEM; S-4800, Hitachi Ltd., Tokyo, Japan) at an accelerating voltage of 10 kV.

**Figure 1:**
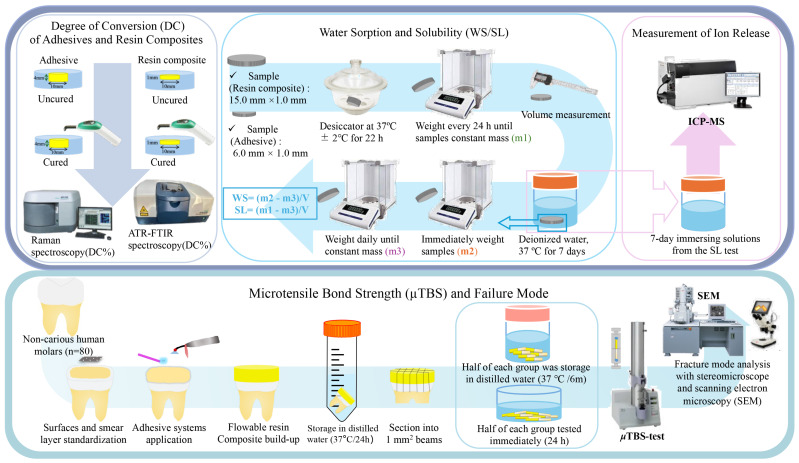
Schematic illustration of sample preparation of adhesives and resin composites for the measurement of degree of conversion, water sorption and solubility, ions release, microtensile bond strength to dentin, and failure mode.

Figure 1 illustrates the adhesives’ and resin composites’ sample preparation for each testing method.

### 2.6. Statistical Analysis

All data were tested for normality using the Shapiro–Wilk and homogeneity of variance using Levene’s tests. The level of significance was set at α = 0.05. The DC (%) data were normally distributed (*p* ≥ 0.05), and the variance was homogenous (*p* ≥ 0.05); therefore, a one-way ANOVA and independent sample *t*-test were performed for the adhesives and resin composites. Post hoc multiple comparisons were performed using the Bonferroni test. As the WS, SL, and ion release date of the adhesives and resin composites were not normally distributed, the Kruskal–Wallis and Mann–Whitney U tests were conducted, followed by post hoc multiple comparisons with a Bonferroni adjustment. Since the µTBS data were normally distributed (*p* = 0.056) and the variance was homogenous (*p* = 0.493), the subsequent analyses were carried out using a three-way ANOVA (factors: adhesives, composites, and storage period) and Sidak post hoc tests. The statistical analysis was conducted using the statistical software package for medical science (SPSS Ver.27 for Windows, SPSS Inc., Chicago, IL, USA).

## 3. Results

### 3.1. Degree of Conversion

**Figure 2:**
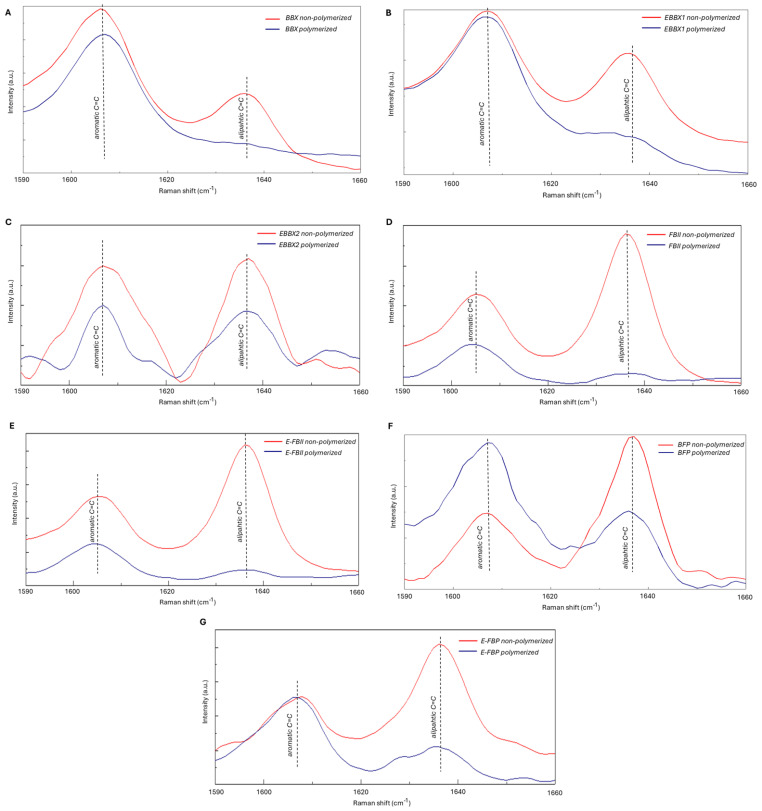
Curve-fitting analysis for average spectra from adhesives and resin composite. Area values for the two reference peaks were calculated: 1608 cm^−1^ (internal standard aromatic carbon double bond, C=C) and 1638 cm^−1^ (methacrylate C=C). (**A**) BBX; BeautiBond Xtreme; (**B**) E-BBX1: Modified Adhesive 1; (**C**) E-BBX2: Modified Adhesive 2; (**D**) FBII: FL-BOND II; (**E**) E-FBII: Silica-containing adhesive; (**F**) BFP: BEAUTIFIL Flow Plus (F00); (**G**) E-BFP: silica containing flowable resin.

Table 2 and Figure 2 describe the DC of adhesive systems and resin composites. A significant difference in the DC of adhesives was observed (*p* = 0.011). E-BBX2 showed the lowest DC (82.63 ± 1.65%), which was inferior to the highest DC presented by BBX (97.22 ± 1.09%). No statistically significant differences were found between the DCs of resin composites (*p* = 0.68).

### 3.2. Water Sorption and Solubility

The WS and SL values (µg/mm^3^) measured for the adhesives and resin composites tested are shown in Table 3. The WS values of all adhesives did not comply with the ISO 4049:2019 threshold (≤40 μg/mm^3^). No significant differences in WS were detected between BBX, E-BBX1, E-BBX2, and FBII (*p* > 0.05). E-FBII produced the lowest WS (64.37 ± 1.86 µg/mm^3^), and this value was significantly lower than those of BBX (165.70 ± 65.03 µg/mm^3^), E-BBX1 (127.58 ± 13.21 µg/mm^3^), and E-BBX2 (150.62 ± 41.13 µg/mm^3^). The SL values of BBX, E-BBX1, and E-BBX2 also did not comply with that ISO requirement (≤7.5 µg/mm^3^). The SL of FBII (4.73 ± 1.76 µg/mm^3^) and E-FBII (4.06 ± 1.41 µg/mm^3^) were significantly lower than those of BBX (71.54 ± 32.86 µg/mm^3^) and E-BBX1 (51.96 ± 13.20 µg/mm^3^) (*p* < 0.05), but they did not show a significant difference compared to E-BBX2 (71.17 ± 9.46 µg/mm^3^). No significant difference in SL was detected among BBX, E-BBX1, and E-BBX2 (*p* > 0.05). The values of WS and SL in both resins complied with the ISO 4049:2019. The WS and SL of BFP (31.29 ± 1.19 µg/mm^3^ and 2.99 ± 0.45 µg/mm^3^) are significantly higher than those of E-BFP (21.3 ± 0.72 and 1.4 ± 0.28 µg/mm^3^) (*p* < 0.05).

### 3.3. Measurement of Ion Release

The results of ion release from each material are shown in Table 4. F ion was only detected in the materials with S-PRG fillers (FBII [0.165 µg/mL] and BFP [0.575 µg/mL]). FBII had a higher B ion release than the other adhesives (*p* < 0.05), and a higher Si ion release than BBX and E-BBX1 (*p* < 0.05). E-BBX1 showed significantly higher Na ion release than BBX (*p* < 0.05). The resin composite BFP released more B, Si, Sr, and F ions than E-BFP (*p* < 0.05). The concentrations of Al ions were all below the detection limit (<0.01 µg/mL).

### 3.4. µTBS and Fracture Mode

The µTBS results are shown in Table 5. A three-way ANOVA revealed significant main effects of adhesive (F = 2.803, *p* = 0.028), storage period (F = 10.113, *p* = 0.002), and composite (F = 18.865, *p* < 0.001) on the bond strength. However, the interaction of these three factors was not statistically significant (F = 1.949; *p* = 0.106). Furthermore, statistically significant interactions were found between storage period and composites (F = 12.764, *p* < 0.001). Simple main effects analyses showed that the 24 h µTBS of BFP used with all adhesives was significantly higher than that of E-BFP (*p* < 0.001), but they did not statistically differ after 6 m of storage (*p* > 0.05). The highest bond strength results were produced when BFP was used to restore the teeth regardless of the adhesive utilized at 24 h testing (*p* < 0.05). The µTBS of BFP utilized with E-BBX1, E-BBX2, and FBII was significantly reduced after 6 m of water storage (*p* < 0.001). Pairwise comparisons showed that at 24 h, E-BBX1 and E-BBX2 restored with BFP [50.61 ± 10.10 MPa and 49.73 ± 7.42 MPa, respectively] showed higher bond strengths than those restored with E-BFP (*p* < 0.001 and *p* = 0.006, respectively).

**Figure 3:**
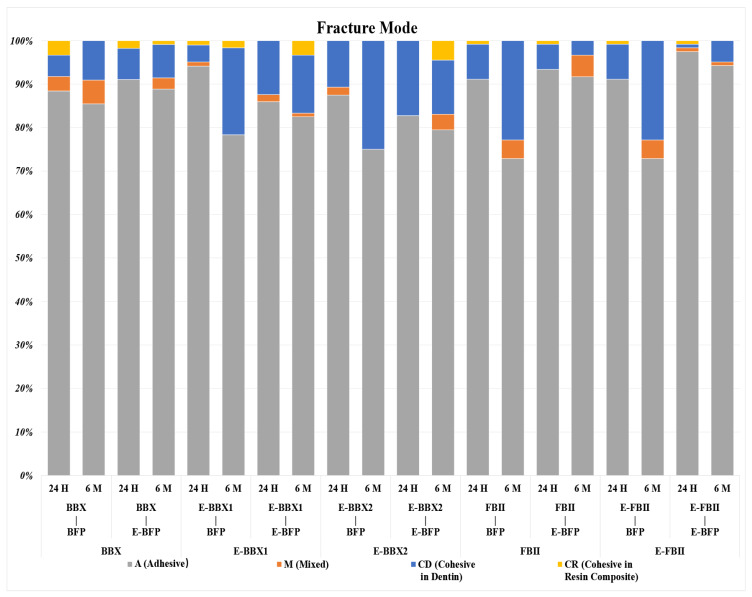
Fracture mode according to materials and storage time. A, adhesive failure; M, mixed failure; CD, cohesive failure in dentin; CR, cohesive failure in resin composite; BBX, BeautiBond Xtreme; E-BBX1, Modified Adhesive 1; E-BBX2, Modified Adhesive 2; FBII, FL-BOND II; E-FBII, silica-containing adhesive; BFP, BEAUTIFIL Flow Plus (F00); E-BFP, silica-containing flowable resin composite.

**Figure 4:**
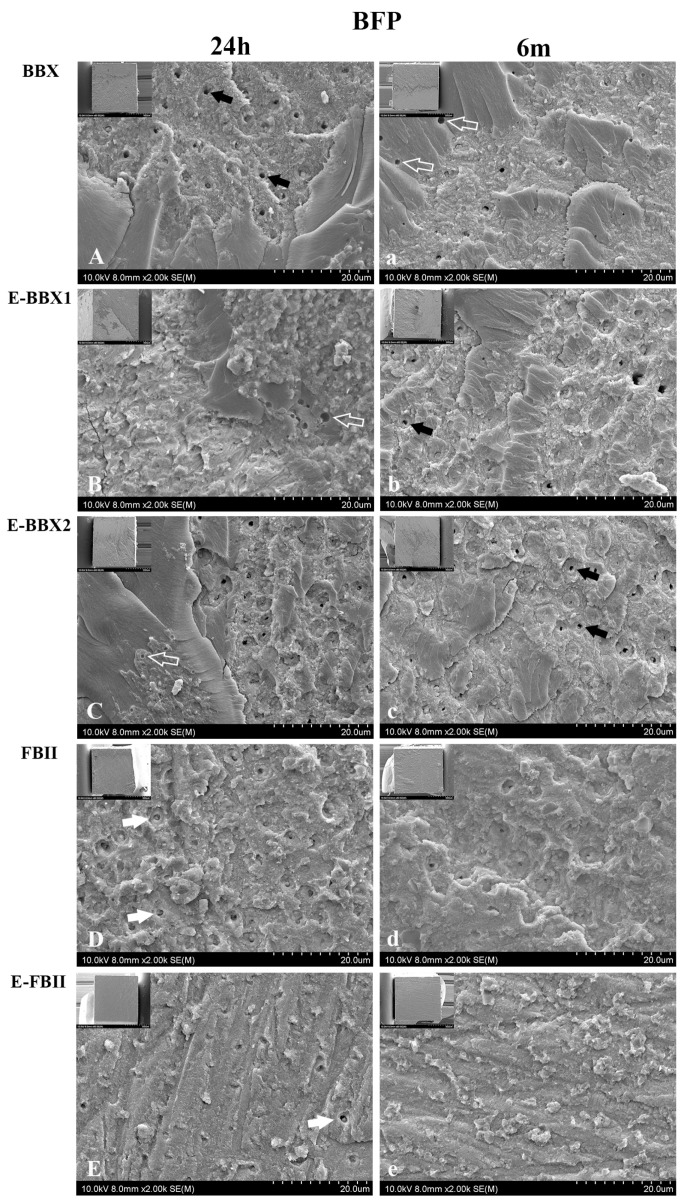
Representative SEM images of each group’s fractured dentin surfaces that were restored with BFP after 24 h (**A**–**E**) and 6 months (**a**–**e**). Each image is magnified 2000×, with the lower magnification (80×) image in the upper left corner. At 24 h, in the BBX, E-BBX1, and E-BBX2 groups, failure occurred within the adhesive layer. Blisters were observed on the fractured surfaces of E-BBX1 and E-BBX2 (empty white arrows). Open dentin tubules could be observed (black arrows). The fracture of FBII and E-FBII groups occurred below the adhesive layer, on the surface of the hybrid layer, and most of the dentin tubule openings were closed (white arrows). Fracture images of all groups of 6 months were similar to those at 24 h. BBX: BeautiBond Xtreme; E-BBX1: Modified Adhesive 1; E-BBX2: Modified Adhesive 2; FBII: FL-BOND II; E-FBII: silica-containing adhesive; BFP: BEAUTIFIL Flow Plus (F00); E-BFP: silica-containing flowable resin composite.

**Figure 5:**
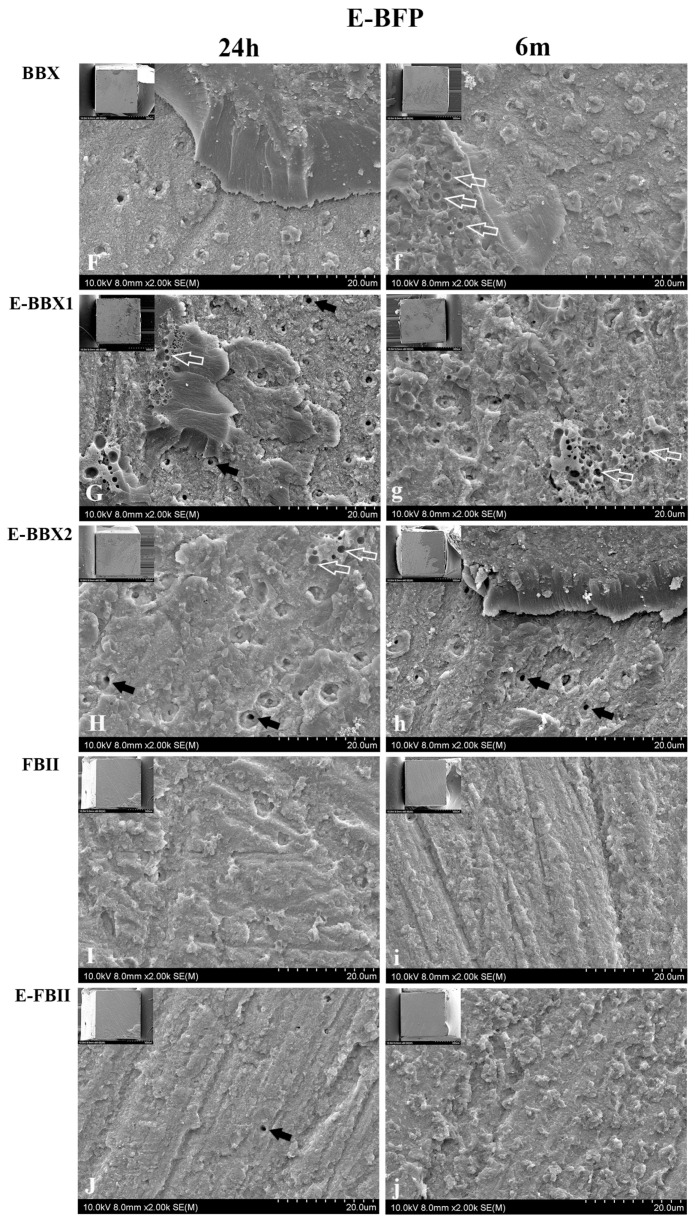
Representative SEM images of each group’s fractured dentin surfaces that were restored with E-BFP after 24 h (**F**–**J**) and 6 months (**f**–**j**). Each image is magnified 2000×, with the lower magnification (80×) image in the upper left corner. At 24 h, in the BBX, E-BBX1, and E-BBX2 groups, failure occurred within the adhesive layer. Open dentin tubules could be observed (black arrows), indicating debonding of adhesives. A greater number and density of blisters are visible at the interface of group E-BBX1 (empty white arrows). The fracture of FBII and E-FBII groups occurred below the adhesive layer, visible traces of the process of preparing the tarnished layer can be seen, and most of the dentin tubule openings were closed. Fracture images of all groups of 6 months (**f**–**j**) were similar to those at 24 h (**F**–**J**), except that BBX exhibited some blisters in the adhesive layer. BBX: BeautiBond Xtreme; E-BBX1: Modified Adhesive 1; E-BBX2: Modified Adhesive 2; FBII: FL-BOND II; E-FBII: silica-containing adhesive; BFP: BEAUTIFIL Flow Plus (F00); E-BFP: silica-containing flowable resin composite.

The percentage of fracture modes is presented in Figure 3, and representative SEM images of adhesive failure restored with BFP and E-BFP are displayed in Figure 4 and Figure 5. Regardless of the storage period, a consistent pattern of adhesive failures was observed for the combinations of different adhesives and resin composites. However, 6 m of water storage did not significantly impact the distribution of failure mode. The three UAs showed adhesive failures with adhesive fragments remaining attached to the dentin (Figure 4A–C,a–c and Figure 5F–H,f–h). The dentin side fractured surfaces of E-BBX1 and E-BBX2 exhibited more circular blisters in the bonding layer (empty white arrows in Figure 4B,C and Figure 5G,g,H) than BBX when utilizing both resin composites, and open dentin tubules could be observed (black arrows). The morphology of the fracture surfaces on the dentin side of the FBII and E-FBII groups exhibited similarities with the use of both resin composites (Figure 4D,E,d,e and Figure 5I,J,i,j); most of the specimens showed complete debonding of the adhesive layer from dentin with exposure of the top of the hybrid layer (Figure 4D,E,d,e and Figure 5I,J,i,j), regardless of the resin composite materials, and dentin open tubules were barely perceptible.

## 4. Discussion

The DC of resin-based materials is influenced by filler ratio, photo-initiators, monomer and co-initiator properties, and light-curing conditions [19,20]. This study supports evidence from previous observations that there was no significant difference in the DC between BFP and other resin composites [21,22]. Thus, the first hypothesis can be rejected based on the DC results. The restorative resin composites in this study were similar in composition, as can be seen in their spectra (Figure 2), except for their inorganic content. Hence, it may be possible to infer that the filler type did not affect the DC of the tested resins. Regarding the adhesives, E-BBX2’s DC was significantly lower than that of BBX, and these results are consistent with previous reports [23]. According to this result, it could be suggested that, as HEMA concentration increased, DC declined [23], as observed in Figure 2 and Table 2. This may be due to the lower polymer reactivity of monomethylated HEMA [5]. Also, the hydrophobic photo-initiators and co-initiators in the UAs might not be compatible with hydrophilic HEMA, resulting in a lower DC [24]. HEMA-free UAs demonstrated a DC compatibility with the 2-SEAs [25], corroborating our findings.

The diffusion process regulates the water sorption and solubility of restorative materials. The absorption of water by the polymer matrix and leaching can lead to the separation of the filler from the matrix or even the deterioration of the fillers through hydrolysis [5]. Typically, water absorption is reduced in materials with a higher filling level but is also affected by the composition of monomers [26]. ISO 4049:2019 recommends that the WS of resin-based materials be ≤40.0 μg/mm^3^, with which both restorative resin composites (i.e., BFP and E-BFP) comply. BFP’s WS agrees with previous findings and is higher than E-BFP, which could be attributed mainly to the S-PRG fillers [22]. The pre-reacted glass-polyacid zones inside the BFP S-PRG filler’s structure may create an osmotic action that causes swelling and pressure [27], which could explain BFP’s WS result. The WS of all adhesives failed to comply with the ISO 4049:2019 threshold. As only the bonding resins of FBII and E-FBII were measured, they resulted in a lower WS than the other UAs. These materials mainly comprise UDMA, which is more hydrophobic than Bis-GMA. This is because Bis-GMA contains more hydrophilic hydroxyl groups of Bis-GMA, which form stronger hydrogen bonds with the water molecules than the urethane groups of UDMA, thus resulting in low WS values [28,29]. The WS of the UAs is influenced by the hydrophilic and acidic functional monomers as well as the composition and concentration of the solvent [5]. One potential reason for the intriguing outcome of HEMA-free BBX displaying a similar WS to E-BBX1 and E-BBX2 is its solvent, acetone. Although BBX is HEMA-free, the high contents of acetone, water, and functional monomer result in high hydrophilicity, like that of the other two HEMA-containing UAs. Similar findings were reported concerning another HEMA-free universal, which showed higher WS than other UAs [25]. The high WS of UAs could be attributed to water bonded to polar regions of the polymer through hydrogen bonds [30]. Regarding the SL, only BFP, E-BFP, FBII, and E-FBII, composed of hydrophobic monomers, complied with the ISO 4049:2019 recommendation (≤7.5 μg/mm^3^). The S-PRG fillers in BFP are more soluble and easily released than those silica fillers contained in E-BFP, thus explaining the higher SL than E-BFP [31]. The SL of BBX, E-BBX1, and E-BBX2 greatly exceeded FBII and E-FBII, coherent with the high content of solvents, hydrophilic and/or ionic monomers in universal adhesives [32,33]. Thus, considering the WS and SL findings, the second null hypothesis was rejected.

Ion release from dental materials is beneficial to stabilizing the collagen matrix, stimulating remineralization, and reinforcing dentin, as depicted in a recent study [34]. In our study, the ion release measurement demonstrated that BFP resin composite exhibited a higher release of B, Si, Sr, and F ions than E-BFP resin, which was corroborated by Shimizu et al. [17]. Nevertheless, in previous investigations, dentin pretreated with S-PRG filler eluent showed greater tensile strength than that treated with NaF [34]. The F, Na, and Sr ions can enhance enamel and dentin’s mechanical strength and acid resistance by forming fluorinated and sodium/strontiated hydroxyapatite [11]. Furthermore, it has been suggested that Sr and F ions could facilitate remineralization and suppress matrix metalloproteinase activity [12]. On the other hand, incorporating boron in the hydroxyapatite structure (mainly as borate substituting phosphate and OH groups) positively impacts dentin by providing antimicrobial protection and promoting remineralization, thereby restoring dentin’s mineral content and structural integrity [11]. A previous study demonstrated that combining the universal adhesive BeautiBond Xtrene with a resin composite containing 70 wt% S-PRG filler increased the hardness of the dentin around the restoration, probably due to the release of B and F ions. [14]. Additionally, FBII, the only adhesive containing S-PRG filler, exhibited a greater B and F ions release than the others. It could be suggested that due to the absence of silica fillers, the three UAs showed lower Si ion release than the other 2-SEA that incorporate fillers. The ion release pattern seen in this study aligns with findings from earlier investigations [11]. The non-detectable release of Al ion was also previously reported [35]. It is relevant to highlight that failure to detect ions released from S-PRG fillers-containing materials does not necessarily mean there is no release, as detection limits immersion liquids, and ratios could impact the ability to detect the actual ions’ leaching [14]. Similar results to the current study were obtained in recent research, where an injectable resin composite containing S-PRG fillers released significantly more B, Sr, and F ions than the silica-filled material, and it was comparable to BFP, possibly [36]. Imazato et al. have demonstrated that materials with S-PRG fillers, such as RC, adhesive, and resin cement, can continue to release ions for more than 1 year, even though the release patterns may vary [11].

The µTBS results indicated that restorative resin composite and storage period significantly influenced the bond strength of the adhesives to dentin; hence, the third null hypothesis was rejected. Regarding the 24 h µTBS, E-BBX1 and E-BBX2 presented higher values when utilizing BFP. This finding accords with earlier observations, which showed that differences in the mechanical properties caused by various compositions of restorative resin composites could influence the µTBS to dentin [37,38]. The critical difference between BFP and E-BFP resin composites is the filler type, as BFP contains S-PRG filler, and E-BFP is loaded with silica. It has been reported that the dentin shear bond strength of Beautifil II, which also contains S-PRG filler, is significantly greater than that of other bioactive restorative materials [39]. This different behavior is attributed to the high concentration of S-PRG filler (83.3 wt%) and the resin’s minimal amount of volumetric shrinkage [39]. In addition, the positive effect of BFP’s S-PRG filler content on E-BBX1 and E-BBX2 might be related to the HEMA content in these universal adhesives, which makes them behave as semi-permeable membranes [1]. Indeed, ions released from S-PRG-filled resins could penetrate UA’s semi-permeable membranes and reach tooth substrates at the cavity wall [40]. This ion-releasing process might prevent microbial leakage into the adhesive interface, contributing to the inhibition of demineralization around resin composite restorations [14,40] and creating more durable bonds [41,42]. Nevertheless, the restorative resin composite did not affect the bond strength of the BBX, which may be attributed to the fact that the absence of HEMA may not have resulted in a further thinning of the interfacial thickness compared to the other two UAs to which HEMA was added [43]. Moreover, strong air-blowing during BBX application might have removed the water at the interface, resulting from the adhesives’ components’ phase-separation and generating few defects in the bonded layer [44].

Conversely, the impact of the restorative resin seems less significant on the bonding capabilities of 2-SEAs compared to UAs. As 2-SEAs, FBII and E-FBII present a separate resin bonding layer, and their overall thickness is higher than that of UAs, which is usually less than 10 µm [1], favoring the absorption of interfacial tension and stress. Most adhesive failures in the FBII and E-FBII showed complete debonding of the adhesive layer from dentin (Figure 4 and Figure 5), which implies that thicker adhesive layers may be more robust and fracture-resistant.

Interestingly, the bond strength of all adhesives to dentin was similar at 24 h when the same resin composite was utilized. The tested UAs presented HEMA concentrations from 0 to 10%, and the role of concentration variation is not precisely correlated with the bonding behavior of adhesives as other components also vary among the adhesives (e.g., solvents, functional monomers, filler content) [30,45]. Low concentrations of HEMA (10%) have been described to increase the 24 h dentin µTBS in one-step SEA, supposedly due to improved wetting properties [46]. Contrarily, UAs’ stable dentin bonds have been reported over time with 2.5–10% HEMA content [45], which follows our findings.

After 6 m of water storage, the µTBS levels of the E-BBX1 and E-BBX2 used with BFP were significantly lower than the 24 h µTBS. Furthermore, there was no difference between BFP and E-BFP after 6 m storage, irrespective of the adhesives. The possible explanation for the 6 m bond strength decrease of E-BBX1 and E-BBX2 with BFP also relies on the higher WS of the BFP compared to the E-BFP and the long-term detrimental effects of HEMA hydrophilicity. Recent research has indicated that resins with S-PRG fillers experience a notable decrease in flexural strength after 30 days of water storage compared to 1 day, likely due to the greater WS [36,47]. A similar result was observed in another investigation when the bond strength of Beautifil II decreased as WS increased during prolonged water storage [48]. Tang et al. (2024) have reported that the presence of HEMA in the UAs led to an increase in WS and a decrease in µTBS after aging. However, the HEMA-free UAs showed no significant changes in WS and µTBS after 50,000 thermocycles, regardless of application mode [30]. Furthermore, a significant reduction in the µTBS of HEMA-containing UAs following thermal cycling was related to the enhanced water permeability resulting from the lower levels of DC and HEMA present [49], consistent with the E-BBX2 results in our study.

The outcomes of this study should be carefully considered, as limitations in its design need to be mentioned. Chemical analyses were not performed on the dentin to determine if passage and deposition of released ions from the composite resin occurred. In addition, to ensure minimal possible variation in components, all tested materials were obtained from the same manufacturer. Hence, subsequent research should add materials from other manufacturers to assess compatibility between different products

## 5. Conclusions

Within the design of this study, commercial adhesives presented the highest degree of conversion. The adhesives did not comply with the water sorption requirements from ISO 4049:2019, and only the bonding resin of the two-step self-etch adhesives complied with the solubility threshold. S-PRG filler-containing resins released more ions than the one containing silica filler only. These findings indicate that S-PRG filler-containing resin composites positively affected the bond strength of the tested universal adhesives, regardless of HEMA concentration after 24 h of storage, comparable to that of the reference two-step adhesives. However, the decrease in the bond strength after 6 months of storage depended on the combination of adhesive and resin composite.

## 6. Clinical Significance

The choice of adhesive and restorative resin composite is significant in preserving the longevity of the restoration, and bioactive S-PRG fillers containing resin materials may stabilize the bonding effect of universal adhesives, especially when the HEMA-free adhesive is utilized.

## Figures and Tables

**Table 1:** Commercial names, batch numbers, abbreviations, compositions, and manufacturer’s instruction of the materials used in the study commercial.

**Adhesives**	**Abbreviations**	**Compositions**	**Manufacturer’s Instructions**
BeautiBond Xtreme (Shofu Inc., Kyoto, Japan, Lot. 122012) (Universal Adhesive)	BBX	Acetone, distilled water, Bis-GMA, carboxylic acid monomer, phosphoric acid monomer, TEGDMA, acid resistant silane coupling agent, others.	Apply bonding adhesive to the dentin surface. Apply gentle air for 3 s and strongly air until dry. Light cure for 5 s.
Modified Adhesive 1 (Shofu Inc., Kyoto, Japan, Lot. 230714) (Universal Adhesive)	E-BBX1	HEMA (5 wt%), acetone, distilled water, Bis-GMA, carboxylic acid monomer, phosphoric acid monomer, TEGDMA, acid resistant silane coupling agent, others.	Apply bonding adhesive to the dentin surface. Apply gentle air for 3 s and strongly air until dry. Light cure for 5 s.
Modified Adhesive 2 (Shofu Inc., Kyoto, Japan, Lot. 230714) (Universal Adhesive)	E-BBX2	HEMA (10 wt%), acetone, distilled water, Bis-GMA, carboxylic acid monomer, phosphoric acid monomer, TEGDMA, acid resistant silane coupling agent, others.	Apply bonding adhesive to the dentin surface. Apply gentle air for 3 s and strongly air until dry. Light cure for 5 s.
FL-BOND II (Shofu Inc., Kyoto, Japan, Lot. 092130) (Two-step, self-etch adhesive)	FBII	Primer: water, ethanol, carboxylic acid monomer, phosphoric acid monomer and initiator Adhesive: SPRG based on fluoroboroaluminosilicate glass, UDMA, TEGDMA, HEMA, polymerization initiator.	Apply primer to the dentin surface and leave for 10 s. Apply gentle air until dry. Apply bonding adhesive to the dentin surface. Light cure for 10 s.
Silica-containing adhesive (Shofu Inc., Kyoto, Japan, Lot. HFLB01) (Two-step, self-etch adhesive)	E-FBII	UDMA, HEMA, TEGDMA, polymerization initiator, silica filler, others Primer: water, ethanol, carboxylic acid monomer, phosphoric acid monomer and initiator. Adhesive: silica filler, UDMA, TEGDMA, HEMA, polymerization initiator, others.	Apply primer to the dentin surface and leave for 10 s. Apply gentle air until dry. Apply bonding adhesive to the dentin surface. Light cure for 10 s.
**Resin Composites**	**Abbreviations**	**Compositions**	**Manufacturer’s Instructions**
BEAUTIFIL Flow Plus F00 (Shofu Dental, Kyoto, Japan, Lot. 122012) (Flowable restorative resin composite)	BFP	Bis-GMA, TEGDMA, S-PRG filler based on fluoroboroaluminosilicate glass, polymerization initiator, pigments, others.	Apply the material in layers with a needle tip to build up a 4 mm thickness resin block. Each layer is no more than 2 mm thick, and light cure for 10 s.
Silica-containing flowable resin composite (Shofu Dental, Kyoto, Japan, Lot. HBFP02) (Flowable restorative resin composite)	E-BFP	Bis-GMA, TEGDMA, silica filler, polymerization initiator, pigments, others.	Apply the material in layers with a needle tip to build up a 4 mm thickness resin block. Each layer is no more than 2 mm thick, and light cure for 10 s.

Abbreviations: HEMA, 2-Hydroxyethyl Methacrylate; Bis-GMA, bisphenol A diglycidyl methacrylate; TEGDMA, triethylene glycol dimethacrylate; S-PRG, surface pre-reacted glass-ionomer; UDMA, urethane dimethacrylate.

**Table 2:** Degree of conversion (DC: %) expressed as Mean (SD) of adhesives systems and resin composites (*n* = 3).

**Adhesive Systems (Abbreviation)**	**DC**
BBX	97.22 (1.09) ^A^
E-BBX1	93.34 (2.96) ^A,B^
E-BBX2	82.63 (1.65) ^B^
FBII	92.87 (5.40) ^A,B^
E-FBII	87.62 (6.41) ^A,B^
**Resin Composites (Abbreviation)**	**DC**
BFP	79.78 (1.27) ^a^
E-BFP	74.72 (3.29) ^a^

Different uppercase letters indicate statistically significant differences between adhesives (*p* < 0.05), and the same lowercase letters refer to statistically significant similarity in resin composites (*p* > 0.05). BBX; BeautiBond Xtreme; E-BBX1: Modified Adhesive 1; E-BBX2: Modified Adhesive 2; FBII: FL-BOND II; E-FBII: Silica-containing adhesive; BFP: BEAUTIFIL Flow Plus (F00); E-BFP: silica containing flowable resin.

**Table 3:** Water sorption (WS) and solubility (SL) expressed as Mean (SD) in (μg/mm^3^) of adhesives systems and resin composites (*n* = 6).

**Adhesive Systems**	**WS**	**SL**
BBX	165.70 (65.03) ^A^	71.54 (32.86) ^A^
E-BBX2	150.62 (41.13) ^A^	71.17 (9.46) ^A^
E-BBX1	127.58 (13.21) ^A^	51.96 (13.20) ^AB^
FBII	81.11 (3.27) ^AB^	4.73 (1.76) ^B^
E-FBII	64.37 (1.86) ^B^	4.06 (1.41) ^B^
**Resin Composites**	**WS**	**SL**
BFP	31.29 (1.19) ^a^	2.99 (0.45) ^a^
E-BFP	21.30 (0.72) ^b^	1.40 (0.28) ^b^

Different uppercase letters in each column indicate significant differences between the adhesive, and different lowercase letters indicate significant difference between resin composites (p < 0.05). BBX; BeautiBond Xtreme; E-BBX1: Modified Adhesive 1; E-BBX2: Modified Adhesive 2; FBII: FL-BOND II; E-FBII: Silica-containing adhesive; BFP: BEAUTIFIL Flow Plus (F00); E-BFP: silica containing flowable resin.

**Table 4:** Ion release results are expressed as median (minimum/maximum) in µg/mL of adhesives systems and resin composites (*n* = 6).

**Adhesive Systems**	**B**	**Na**	**Si**	**Sr**	**F**
BBX	1.60000 ^B^ (1.45000/2.64000)	2.06000 ^B^ (1.67000/3.51000)	0.08700 ^C^ (0.07000/0.20200)	0.00325 (0.00060/0.00350)	<detection limit ^B^
E-BBX1	2.35000 ^B^ (2.06000/2.55000)	3.27000 ^A^ (2.89000/3.68000)	0.11950 ^BC^ (0.08300/0.17600)	0.00390 (0.00240/0.00450)	<detection limit ^B^
E-BBX2	2.22500 ^B^ (1.81000/2.83000)	3.07500 ^A^ (2.64000/4.01000)	0.13750 ^ABC^ (0.10300/0.47300)	0.00285 (0.00260/0.00320)	<detection limit ^B^
FBII	3.41500 ^A^ (1.96000/4.04000)	2.67500 ^AB^ (2.38000/3.20000)	0.55500 ^A^ (0.47600/0.84500)	0.01005 (<detection limit/0.12000)	0.16500 ^A^ (<detection limit/0.27000)
E-FBII	1.72000 ^B^ (1.37000/2.97000)	2.53000 ^AB^ (2.29000/3.25000)	0.40950 ^AB^ (0.35200/0.70400)	0.00005 (<detection limit/0.10800)	<detection limit ^B^
**Resin** **Composites**	**B**	**Na**	**Si**	**Sr**	**F**
BFP	3.53500 ^a^ (3.1300/3.8300)	2.66000 (2.32000/3.31000)	0.46300 ^a^ (0.30800/0.76300)	0.75900 ^a^ (0.32600/1.53000)	0.57500 ^a^ (0.30000/1.00000)
E-BFP	1.57500 ^b^ (0.60500/1.94000)	2.24500 (0.87900/2.74000)	0.25300 ^b^ (0.12500/0.43600)	0.00205 ^b^ (0.00100/0.00280)	<detection limit ^b^

The concentrations of Al ions were all below the detection limit (<0.01 µg/mL). Different uppercase/lowercase letters in each column indicate significant differences among the adhesives/resin composites (*p* < 0.05). BBX; BeautiBond Xtreme; E-BBX1: Modified Adhesive 1; E-BBX2: Modified Adhesive 2; FBII: FL-BOND II; E-FBII: Silica-containing adhesive; BFP: BEAUTIFIL Flow Plus (F00); E-BFP: silica containing flowable resin.

**Table 5:** Microtensile bond strength (µTBS) is expressed as Mean (SD) in MPa (*n* = 8). For each adhesive system, two different restorative resin composites were tested after 24 h and 6 months of storage in distilled water.

Adhesive System	Resin Composite	24 h	6 Months
BBX	BFP	42.37 (4.60) ^Aa1^	41.72 (3.24) ^Aa1^
	E-BFP	35.38 (4.12) ^Aa1^	35.87 (11.19) ^Aa1^
E-BBX1	BFP	50.61 (10.10) ^Aa1^	39.65 (6.66) ^Ba1^
	E-BFP	36.15 (5.04) ^Ab1^	41.39 (9.86) ^Aa1^
E-BBX2	BFP	49.73 (7.42) ^Aa1^	40.17 (7.32) ^Ba1^
	E-BFP	39.91 (4.01) ^Ab1^	39.59 (4.94) ^Aa1^
FBII	BFP	50.60 (8.41) ^Aa1^	35.35 (5.02) ^Ba1^
	E-BFP	44.24 (5.42) ^Aa1^	41.99 (5.59) ^Aa1^
E-FBII	BFP	42.55 (8.04) ^Aa1^	41.06 (8.71) ^Aa1^
	E-BFP	35.73 (6.42) ^Aa1^	34.77 (8.87) ^Aa1^

Different uppercase letters indicate significant differences between 24 h and 6 m) for the same adhesive-resin combination (*p* < 0.05); different lowercase letters indicate significant differences between resin composites within each column (24 h or 6 m) for the same adhesive (*p* < 0.05); different numbers indicate significant differences between adhesives within each column for the same resin composite (*p* < 0.05). BBX; BeautiBond Xtreme; E-BBX1: Modified Adhesive 1; E-BBX2: Modified Adhesive 2; FBII: FL-BOND II; E-FBII: Silica-containing adhesive; BFP: BEAUTIFIL Flow Plus (F00); E-BFP: silica containing flowable resin.

## Data Availability

The original contributions presented in the study are included in the article, further inquiries can be directed to the corresponding author.
